# Effects of antioxidants and phosphorylated tau protein on mitochondrial respiration and hydrogen peroxide production

**DOI:** 10.1192/j.eurpsy.2025.1734

**Published:** 2025-08-26

**Authors:** J. Hroudová, Z. Fišar

**Affiliations:** 1 Department of Psychiatry, First Faculty of Medicine, Charles University and General University Hospital in Prague, Prague, Czech Republic

## Abstract

**Introduction:**

Alzheimer’s disease (AD) is a progressive neurodegenerative disease and the most common form of dementia; currently there is no effective causal treatment for AD. The main targets of new drugs for AD are processes related to amyloid beta and tau neurotoxicity, neurotransmission, inflammation, metabolism and bioenergetics, synaptic plasticity, and oxidative stress.

**Objectives:**

The effects of methylene blue, curcumin, ginkgolide B, and melatonin are being tested as antioxidants, tau-aggregation inhibitors, and mitochondrial-targeted therapies. These antioxidants will also be tested as protective agents against tau-induced mitochondrial dysfunction caused by oligomers of phosphorylated tau (P-tau). The aim of our study is to systematically investigate tau-induced mitochondrial dysfunction in isolated brain mitochondria and the protective effects of AD drugs, adjuvants, against mitochondrial dysfunction.

**Methods:**

Isolated pig brain mitochondria were used as an *in vitro* biological model to evaluate the effects of antioxidants. Hydrogen peroxide formation was determined by fluorescence measurements (starting with Amplex Red, horseradish peroxidase). Mitochondrial respiration was measured using high-resolution respirometry. The effects of tested drugs were measured at concentrations in the range 0.1 to 500 μmol/l; the effect of P-tau was measured in the range 3-120 nmol/l.

**Results:**

Methylene blue and curcumin significantly reduced hydrogen peroxide production at high concentrations. The antioxidants were further tested in combination with effect of P-tau oligomers in various states of mitochondrial respiration.

**Conclusions:**

The results of this study will contribute to the connection of today’s most accepted hypotheses of AD (amyloid, tau and mitochondrial), to the understanding of intracellular processes associated with the formation and progression of AD. The applied impact of the study will be the introduction of an *in vitro* mitochondrial assay to evaluate the anti-tau effects of new AD drugs, which will allow the selection of agents with high potential for causal treatment of AD.

**Disclosure of Interest:**

J. Hroudová Grant / Research support from: Supported by Ministry of Health of the Czech Republic, NU23-04-00032, Z. Fišar Grant / Research support from: Supported by Ministry of Health of the Czech Republic, NU23-04-00032.

